# Pharmaceutical Transformation Products Formed by Ozonation—Does Degradation Occur?

**DOI:** 10.3390/molecules28031227

**Published:** 2023-01-26

**Authors:** Adi Zilberman, Igal Gozlan, Dror Avisar

**Affiliations:** The Hydrochemistry Research Group, The Water Research Center, Porter School of the Environment and Earth Sciences, Faculty of Exact Sciences, Tel Aviv University, Tel Aviv 69978, Israel

**Keywords:** pharmaceutical, ozonation, degradation, transformation product, persistent compound

## Abstract

The efficiency of an advanced oxidation process (AOP) using direct and indirect ozonation for the removal of pharmaceutical residues from deliberately spiked deionized water was examined. Both direct and indirect ozonation demonstrated 34% to 100% removal of the parent compounds. However, based on the products’ chemical structure and toxicity, we suggest that despite using accepted and affordable ozone and radical concentrations, the six parent compounds were not fully degraded, but merely transformed into 25 new intermediate products. The transformation products (TPs) differed slightly in structure but were mostly similar to their parent compounds in their persistence, stability and toxicity; a few of the TPs were found to be even more toxic than their parent compounds. Therefore, an additional treatment is required to improve and upgrade the traditional AOP toward degradation and removal of both parent compounds and their TPs for safer release into the environment.

## 1. Introduction

Domestic wastewater treatment based on conventional activated sludge does not remove persistent micropollutants, such as pharmaceutical compounds (PCs) [1,2,3] an additional treatment is required. One such thoroughly studied treatment is the advanced oxidation process (AOP)-based ozonation [4,5]. PC degradation by ozonation processes has been reported to be extremely efficient. Hansen et al. (2016) [6] reported ozonation removal of 90% for 33 chosen PCs. Lin et al. (2016) [7] investigated the occurrence and removal of 39 pharmaceutical and personal care products (PPCPs) by ozonation; whereas 14 of them were completely removed, one of the studied PCs, sulfamethoxazole (SMX), presented 92% degradation. Paucar et al. (2019) [8] also suggested that ozonation can remove a wide range of PPCPs from secondary effluent. Moreover, the infusion of hydrogen peroxide (H_2_O_2_) into ozonation increased the percent removal of organic and inorganic substances [9]. Von Gunten (2003) [10] demonstrated 50% removal of para-chlorobenzoic acid in groundwater with the addition of H_2_O_2_, as opposed to 20% removal with only ozonation. However, the term “removal” usually refers to degradation of the parent compound without thoroughly addressing the obtained products. Are the latter more biodegradable? Are they less toxic? Are they indeed chemically different from their parent compounds? Are they less persistent? Many studies focusing on one or several compounds have indicated the formation of “degradation products” that are correlated and related only to “parent compound removal” during ozonation. The main purpose of this study was to evaluate the degradation of six representative parent compounds, determine the transformation products (TPs) generated by ozonation and answer the important question of whether degradation has really occurred.

## 2. Results and Discussion

### 2.1. Ozone and Ozone/H_2_O_2_

**Figure 1:**
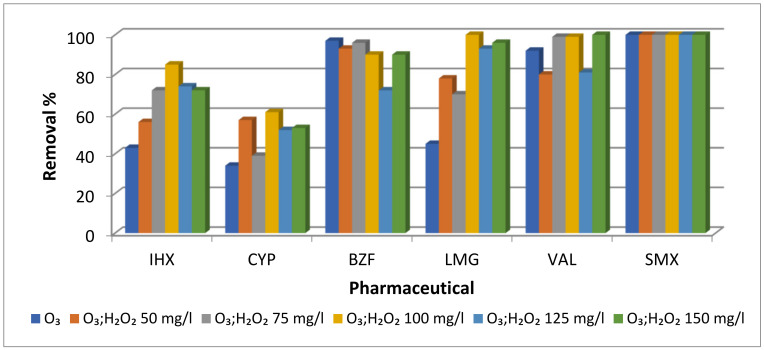
Removal percentages of the six pharmaceutical compounds (PCs) by direct ozonation and indirect ozonation with varying amounts of H_2_O_2_ after 20 min. PCs: iohexol (IHX), bezafibrate (BZF), lamotrigine (LMG), valsartan (VAL), cyclophosphamide (CYP) and sulfamethoxazole (SMX).

The efficiency of indirect versus direct ozonation was evaluated for degradation of the six selected PCs. In addition, the kinetics of indirect ozonation was tested with changing concentrations of H_2_O_2_ ranging from 0.05 mg/L to 0.15 mg/L, to determine the dosage of H_2_O_2_ providing the best removal percentages (Figure 1). SMX, BZF and VAL were highly susceptible to ozonation with almost no change between the direct and indirect procedures. For IHX, CYP and LMG, better removal percentages were obtained with the indirect procedure, and best removal efficiency was achieved with a concentration of 0.1 mg/L of H_2_O_2_. Although the removal appeared to be between 35% and 100% for all of the PCs, the results indicated that neither direct nor indirect ozonation processes actually degraded or removed the parent molecules, as no mineralization was observed by the TOC measurements; however, ozonation did transform those compounds to create TPs. Von Gunten’s (2018) [11] claim that three aspects of TPs should be considered when applying AOP: (i) oxidation treatment leads to a loss of the parent compound’s primary biological activity; (ii) the TPs of the biologically active molecules could potentially be more toxic than the parent compound; (iii) toxic compounds can form from compounds with low biological activity. Therefore, instead of using the term “degradation” as in most studies, the term “transformation” is adopted herein. Transformation means that the parent molecules are not broken down into more biodegradable products, but generally form similar molecules, which differ slightly in structure, but have similar persistence, stability and toxicity.

### 2.2. TPs of the PCs

Oxidizing the PCs with ozone created TPs, without any degradation or mineralization. It is important to emphasize that the mass balances of all the tested PCs and their TPs were not balanced, similar to other studies [12,13,14,15], thus we cannot rule out the possibility that other compounds were created through different pathways but were not detected by the LC or gas chromatography techniques.

**Figure 2:**
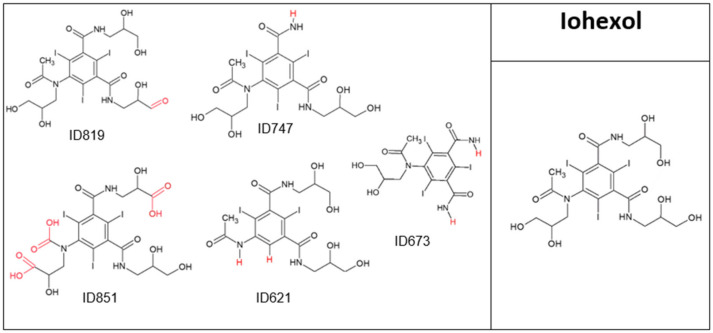
Iohexol and its major transformation products.

IHX (parent compound) concentration decreased by 40% after 20 min of direct oxidation (Figure 1) and five major TPs were obtained (Figure 2); all the TPs retained their aromatic ring, amide groups and the parent molecule’s general structure. TOC results for IHX (Table 1) demonstrated that neither mineralization nor degradation had occurred, supporting the premise of TP formation. Moreover, UV absorbance at 254 nm (A254) measured before and after direct ozonation showed a 75% rise in the absorbance reading, indicating an increase in molecular chromophore groups. IHX has been found to be the iodine source for the formation of chloraminated iodo-trihalomethane and iodo-acid disinfection by-products (DBPs) in chlorinated drinking water. Both by-products are highly genotoxic and cytotoxic in mammalian cells [16]. Furthermore, ozonation of IHX in the presence of bromide enhances DBP formation [17]. Ecosar acute toxicity results showed increased toxicity of the TPs to fish and Daphnia compared to the parent compound, except ID851, which showed decreased toxicity (Table S1 in Supplementary Materials). In addition, ChV values for fish increased dramatically for the TPs ID621 and ID673, 2.5- and 18-fold, respectively, compared to that of the parent compound. The results in Figure 2 and Table 2 provide more strong and solid evidence of molecule transformation, rather than degradation All three iodines remained in four of the TPs, whereas ID621 retained only two iodines; all TPs therefore remained as sources of iodine, meaning that they could continue to be potentially genotoxic and cytotoxic through their persistence in the water cycle.

**Figure 3:**
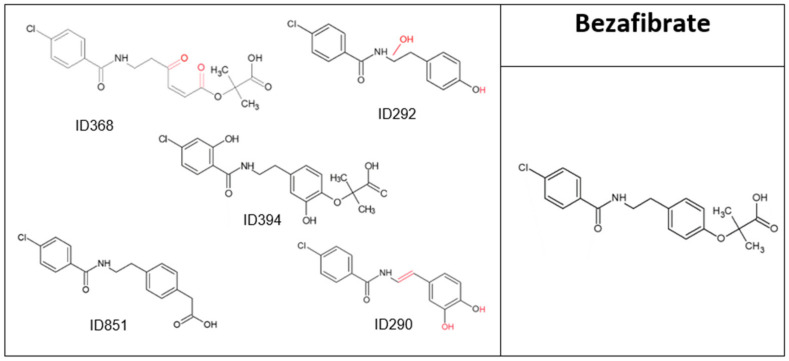
Bezafibrate and its major transformation products.

BZF concentration decreased by 98% after 20 min of direct oxidation (Figure 1), due to its high reaction rate (*K*O_3_ = 590 M^−1^ s^−1^) [21], giving five major TPs (Figure 3 and Table 3); four retained their two aromatic rings and carbonyl groups, while in TP ID368, one aromatic ring was opened by further oxidation. TOC results for BZF before and after (Table 1) showed a decrease of 27%. Although some mineralization occurred, the parent compound had almost completely disappeared; thus, most of the drug did not undergo full mineralization, indicating that non-biodegradable TPs, which still retain most of their parent molecule’s structure, were formed by ozonation. In addition, A254 values tripled after ozonation. BZF is frequently found in wastewater, surface water, groundwater and even drinking water, in some cases reaching concentrations of several micrograms per liter [22,23]; at 57 ng/L, BZF has been reported to damage human sperm DNA [24]. Moreover, the TP ID292 has been reported to be 2–6 times more toxic to algae, Daphnia (acute and chronic toxicity) and fish (chronic toxicity) than the parent compound [25], meaning that the occurrence of TPs does not represent degradation of the parent compound, but a similar compound that could be even more toxic and chemically stable. Ecosar acute toxicity results also showed a 3- to 8-fold increase in toxicity levels (for fish and Daphnia) compared to the parent compound for all of the TPs except ID368, which showed decreased toxicity (Table S2). ChV values in fish increased dramatically for all the TPs, ranging from 3 to 19 times higher than the parent compound toxicity levels. 

**Figure 4:**
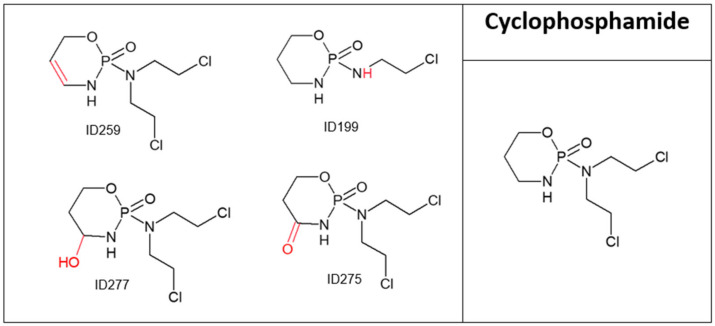
Cyclophosphamide and its major transformation products.

CYP concentration decreased by 36% after 20 min of direct ozonation (Figure 1), and four major TPs were obtained (Figure 4 and Table 4); three presented minor changes compared to the parent compound, whereas TP ID199 showed more considerable changes. TOC results (Table 1) for CYP before and after ozonation showed that neither mineralization degradation had occurred, even though the parent compound decreased by 36%; thus, most of the drug did not undergo mineralization, as supported by the A254 values. CYP has very weak chromophores absorbing at 254 nm; therefore, UV absorbance for the drug itself is very low, but after direct oxidation, an increase in chromophores is observed. Results showed an increase from 1 mAU to 29 mAU before and after direct oxidation, respectively. CYP is an anticancer drug known for being mutagenic, genotoxic and teratogenic [32,33]. Its TPs ID275 and ID277 have been reported as mutagenic, and as harmful to aquatic organisms [34]. Furthermore, TP ID259 was reported to be more toxic than its parent compound. Ecosar acute toxicity results also showed that CYP and all of its TPs are very toxic to *Daphnia* as well as harmful to fish. ChV results for fish showed that CYP and all of its TPs are very toxic, confirming the environmental and health hazards posed by both the parent compound and its TPs (Table S3).

**Figure 5:**
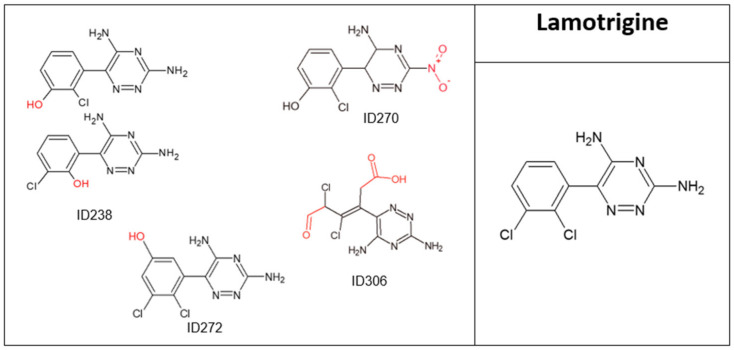
Lamotrigine and its major transformation products.

LMG concentration decreased by 48% after 20 min of direct oxidation (Figure 1). Four TPs were identified (Figure 5 and Table 5); three retained their two aromatic rings, while the TP ID306 had an open benzene ring. TOC results for LMG before and after ozonation (Table 1) showed that neither mineralization nor degradation had occurred, even though the parent compound decreased by 48%; thus, most of the drug did not undergo mineralization. In addition, A254 doubled after ozonation. According to previous studies and Ecosar, LMG is neither toxic nor harmful [37] Nevertheless, all of its major TPs were toxic or harmful to fish and *Daphnia*, while their ChV values for fish were classified as very toxic, with 7 to 33 times higher toxicity levels compared to the parent compound (Table S4). LMG is resistant to advanced treatments such as ozonation and photolysis; it is also very stable in the environment and not susceptible to conventional water treatment processes, and is therefore persistent in the water cycle [38]. Although LMG by itself is not harmful, in the presence of carbamazepine, it is taken up by plants at a higher rate; it was also suggested that exposure to a mixture of PCs, even at low concentrations, could induce its transformation due to enzymatic activation [39].

**Figure 6:**
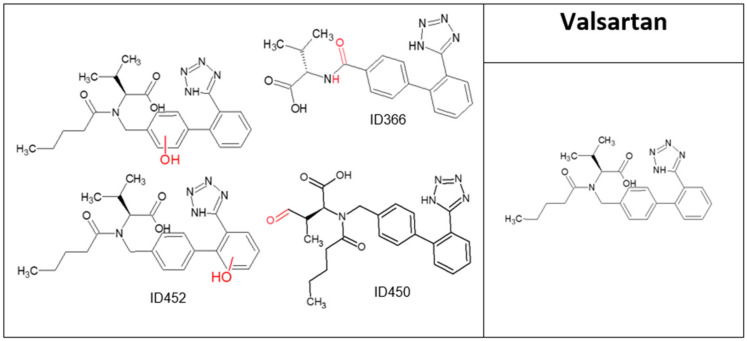
Valsartan and its major transformation products.

VAL concentration decreased by 92% after 20 min of direct ozonation (Figure 1) due to its relatively high reaction rate (*K*O_3_ = 38 M^−1^ s^−1^, (Y. Lee et al., 2014)). Four major TPs were identified (Figure 6 and Table 6), all of which retained their aromatic rings and carbonyl groups. TOC results (Table 1) for VAL before and after ozonation showed a small decrease of 3%, indicating that neither mineralization nor degradation had occurred, even though levels of the parent compound decreased by almost 92%. In addition, A254 increased by 13% after direct oxidation. These results suggest a decrease in TP biodegradability and indicate that the TPs are more persistent than their parent molecule. The TPs ID366 and ID450 demonstrated decreased levels of toxicity compared to the parent compound, both in previous studies [40] and by Ecosar; the latter showed that TPs ID336 and ID452 are more toxic than the parent compound. Both were found harmful to fish and *Daphnia* and to be very toxic to fish with long exposure (Table S5). The influence of combinations of the other compounds and their TPs with VAL and its TPs is unknown and requires further research [40].

**Figure 7:**
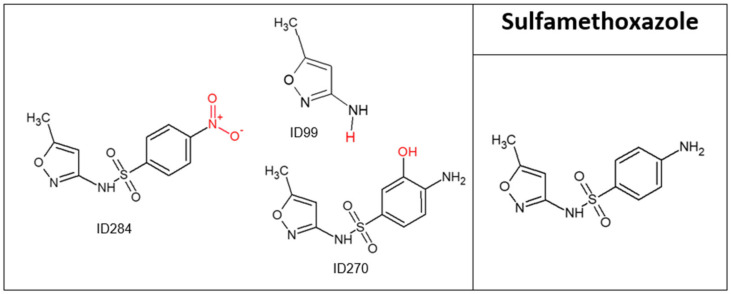
Sulfamethoxazole and its major transformation products.

SMX has a very high reaction rate (*K*O_3_ = 4.7 × 10^4^/5.7 × 10^5^ [41,42] and its concentration decreased by 100% under 3 min of ozonation (Figure 1). Three major TPs were obtained (Figure 7 and Table 7); TP ID99 lost a substantial part of the parent compound and was actually degraded to a non-biodegradable degradation product, while the other two TPs had only minor structural changes. TPs formed and then decreased to undetectable levels after 20 min of oxidation. TOC results (Table 1) for SMX before and after ozonation showed a decrease of 14%. Although some mineralization occurred, the parent compound completely disappeared, and therefore most of the drug did not undergo mineralization, indicating that the non-biodegradable TPs, which still contained most of the parent molecule’s structure, were formed by ozonation. Moreover, SMX was the only drug that showed a decrease in A254 after ozonation, by 28%. Thus, SMX demonstrated a decrease in chromophores, which is in agreement with its transformation product (ID99). Despite these results, the chemical structure of the SMX TPs still included aromatic rings and a sulfamide group, indicating their non-biodegradability and chemical stability. SMX is an antibiotic that has been reported to induce both cytotoxic and chromosomal damage in cultured human lymphocytes [36]. Both SMX and its TPs have been reported to be toxic to *Daphnia magna* (acute) and *Pseudokirchneriella subcapitata* (chronic)*,* with the TPs after ozone treatment showing greater toxicity [43]. The Ecosar results supported this and indicated that TP ID270 is 7 times more toxic than the parent compound (acute) and 18 times more toxic to fish (chronic) (Table S6).

TPs of five of the six studied PCs demonstrated an increase in chromophores and therefore an increase in double-bond conjugation (based on A254). This implies that the formed molecules are more stable, and therefore more persistent than their parent molecule, suggesting a decrease in TP biodegradability.

### 2.3. Summary

The efficiency of AOP-based ozone for the degradation of six selected PCs was evaluated with and without H_2_O_2_. The ozone and radical concentrations used in the study were well accepted (~1 mg ozone/1 mg DOC (dissolved organic carbon)) and affordable.

The obtained LC-MS results indeed showed a decrease in the parent compound, but also the formation of molecules that were chemically similar to the parent compounds, i.e., TPs. The results were supported by LC-MS, TOC, A254 and Ecosar toxicity analysis. From the 6 studied PCs, 25 major TPs were identified and quantified; similarly, Gulde et al. (2021) [46] found 153 new signals, which they identified and associated to 84 TPs and 40 micropollutants after ozonation treatment. From a mass balance perspective, only one-sixth of the mass of the original six PCs was accounted for by the obtained TPs, while the rest remained unaccounted for. The formation of 25 new molecules from 6 molecules should raise a big question regarding the AOP treatment, especially because the TOC results showed only minor mineralization through a decrease in organic carbon for some of the PCs (SMX, BZF, VAL), and no mineralization at all for the others (CYP, IHX, LMG). The UV absorbance of five out of the six PCs demonstrated an increase in aromaticity. Furthermore, for each of the studied PCs, a few or all of their major TPs had higher toxicity levels than their respective parent compounds, posing a greater risk to the environment and to the health of many organisms. Further research is needed to assess the affiliation of the studied PCs’ toxicity to indirect processes, such as enzymatic activation, as suggested for LMG [39]. According to these results, AOP treatment-based ozonation (direct and indirect) should be reassessed, especially where biological treatment combined with activated carbon treatment after ozonation are not sufficient [46,47]. The same applies to the use of a catalyst as a major or only treatment for eliminating pharmaceutical residues from the aquatic environment [9]. The implementation of traditional AOP treatment mostly produces TPs that are characterized as persistent and toxic, calling for the upgrading and improvement of these treatments.

## 3. Materials and Methods

### 3.1. Standards and Reagents

Iohexol (IHX), bezafibrate (BZF), lamotrigine (LMG), valsartan (VAL), cyclophosphamide (CYP) and sulfamethoxazole (SMX) were obtained from Merck-Sigma (Israel). The solvents were LC-grade. Methanol and formic acid were purchased from Bio-Lab (Israel). Individual stock solutions of each compound at a concentration of 100 mg/L were prepared by dissolving the compound in deionized (DI) water. The six PCs were selected based on their presence in Tel Hashomer hospital wastewater and their ubiquity in treated wastewater in Israel, which represent different chemical classes, considered as toxic to various organisms, and have different oxidation reaction rates with ozone and OH, as specified in Table 8.

### 3.2. Laboratory Scale Ozone Experiments 

Laboratory scale ozone experiments were conducted using a 500 mL batch scale cylindrical glass reactor, containing 200 mL at 1 mg/L of each drug, separately, mixed with DI water at a starting pH of 7.0. A laboratory ozone generator (OEM Collecting Industrial, Shenzhen Guangdong, China) was used; the oxygen source was atmospheric air, introduced through the air inlet. A diffuser connected to the ozone generator via PVC tubing bubbled ozone with an output of 200 mg/h at a flow rate of 2.5 L/min for 20 min. The residual ozone concentration was evaluated using the indigo colorimetric method at 5.6 mg/L [51].

### 3.3. Analytical Measurements

Total organic carbon (TOC) method was used to measure and evaluate levels of mineralization. TOC was measured in an Aurora TOC Analyzer (O.I. Analytical, Texas, USA). The instrument measures TOC by acidifying and oxidizing the organic molecule’s carbon in the sample solution into CO_2_ and calculating its concentration using a calibrated infrared reading detector. 

Chromatographic analysis was performed by high-pressure liquid chromatography (HPLC; Agilent 1100 series, CA, USA) equipped with a Synergi polar RP 2.1 × 250 mm, 4 µm analytical column (Phenomenex, CA, USA) at 50 °C. The flow rate was 0.45 mL/min with injection volume of 100 µL. The mobile phase contained 0.1% formic acid (Merck, MO, USA) in water (solution A) and 0.1% formic acid in methanol (solution B). A gradient program was implemented, starting from 0–1 min (hold at 5% solution B), 1–14 min (to 90% solution B), 14–18 min (hold at 90% solution B), 18–21 min (change back to 5% solution B) and 21–28 min (hold at 5% solution B for column equilibration). Detection and quantification were carried out using high-resolution mass spectrometry (HRMS) (Q-TOF Premier, Waters, MA, USA) via an electrospray ionization interface in positive mode. Data were assessed using Waters chromatography Mass Lynx software (v4.1). The selected PCs were identified according to their retention time and molecular ion exact mass [MH]^+^ (Table 9).

### 3.4. Transformation Products’ Identification

The TP were identified and their molecular structures were confirmed according to numerous parameters. The molecular weight was determined by obtaining the [M+H]+ mass by the LC/MS technique, some time by the combination of [M+H]+ and [M+Na]+. The empirical formula was determined by using the high-resolution mass spectrum capacity of the used Q-TOF LC/MS. In addition, as indicated in Table 2, Table 3, Table 4, Table 5, Table 6 and Table 7, the identification of the molecular structures of the TP was based on expected molecular structures, obtained, as a result of the oxidation process of the parent molecules, accompanied and supported also by the literature.

### 3.5. Toxicity Assessment Using the Ecological Structure–Activity Relationships Program (Ecosar 2.0)

Ecotoxicity of the PCs and their TPs was evaluated using Ecosar 2.0. This program uses a computerized system to predict the acute and chronic toxicity of a pollutant to aquatic organisms through structure–activity relationships (SARs). Acute toxicity is measured as LC50, representing the concentration of the toxic substance that causes 50% mortality in fish and Daphnia after 96 h and 48 h exposure, respectively. Chronic toxicity (ChV) in fish assesses the effect of long-term exposure to a pollutant on the organisms. The classifications are very toxic (LC50/EC50/ChV ≤ 1), toxic (LC50/EC50/ChV ≤ 10), harmful (LC50/EC50/ChV ≤ 100) and not harmful (LC50/EC50/ChV > 100) [52].

## 4. Conclusions

Traditional direct and indirect AOP is insufficient for persistent compound degradation and/or mineralization. Results indicated that TPs are obtained, therefore, degradation or mineralization of the persistent parent compounds is not achieved. The detected TPs are more toxic, chemically similar (still contain the toxic moiety) and less biodegradable than parent compounds, meaning that no degradation was obtained. Upgraded and more efficient AOP technologies are needed for the full degradation/mineralization of persistent compounds detected in wastewater and effluent.

## Figures and Tables

**Table 1:** Pharmaceutical compounds TOC and absorbance at 254 nm.

	IHX	CYP	BZF	LMG	VAL	SMX
TOC [ppm] Ozonation at t = 0 min	5.31	4.51	4.43	4.66	6.11	5.40
TOC [ppm] Ozonation at t = 60 min	5.26	4.59	3.16	4.65	5.93	4.66
Abs [mAU] at 254 nm for O_3_ t = 0 min	34	1	10	21	33	50
Abs [mAU] at 254 nm for O_3_ t = 20 min	60	29	33	41	37	36

IHX, iohexol; BZF, bezafibrate; LMG, lamotrigine; VAL, valsartan; CYP, cyclophosphamide; SMX, sulfamethoxazole.

**Table 2:** Iohexol transformation products’ identification information.

N^o^	Rt [min]	[M+H]^+^ [Da]			Formula	Ref.	Suggested Occurrence
		Obtained Mass	Calculated Mass	Deviation (ppm)			
Iohexol (IHX)	4.91, 6.01	821.8934	821.8876	−7.1	C_19_H_26_I_3_N_3_O_9_	[18,19,20]	
ID851	4.26, 4.8	851.8254	851.8253	0.0	C_18_H_20_I_3_N_3_O_12_		Oxidation of three OH groups to obtain three carboxylic acid
ID621	4.45	621.9617	621.9541	−12.1	C_16_H_21_I_2_N_3_O_7_	[18]	Elimination of dihydroxypropyl group Elimination of iodine atom
ID819	5.92, 6.34	819.8809	819.8719	−11.0	C_19_H_24_I_3_N_3_O_9_	[18]	Oxidation of OH group to aldehyde
ID747	6.35	747.8533	747.8508	−3.3	C_16_H_20_I_3_N_3_O_7_	[18]	Elimination of dihydroxypropyl group
ID673	7.32	673.8154	673.8140	−2.1	C_13_H_14_I_3_N_3_O_5_	[18]	Elimination of two dihydroxypropyl groups

**Table 3:** Bezafibrate transformation products’ identification information.

N^o^	Rt [min]	[M+H]^+^ [Da]			Formula	Ref.	Suggested Occurrence
		Obtained Mass	Calculated Mass	Deviation (ppm)			
Bezafibrate (BZF)	15.37	362.1151	362.1154	0.7	C_19_H_20_ClNO_4_	[26,27,28]	
ID292	12.89	292.0708	292.0735	9.2	C_15_H_14_ClNO_3_	[25,29,30]	Elimination of 2-methylpropanoic acid group and hydroxylation of aliphatic group
ID368	14.49	368.0883	368.0895	3.4	C_17_H_18_ClNO_6_	[31]	Two steps: Two hydroxylation of aromatic ring (ortho position) Obtains two carbonyl groups after bond cleavage
ID290	14.82	290.0579	290.0578	−0.2	C_15_H_12_ClNO_3_	[25]	Elimination of H_2_O from ID292 to obtain double bond Hydroxylation of aromatic ring
ID394	14.91	394.1005	394.1052	11.9	C_19_H_20_ClNO_6_	[25,29,30]	Hydroxylation of chloroaromatic ring
ID318	15.59	318.0895	318.0891	−1.3	C_17_H_16_ClNO_3_		

**Table 4:** Cyclophosphamide transformation products’ identification information.

N^o^	Rt [min]	[M+H]^+^ [Da]			Formula	Ref.	Suggested Occurrence
		Obtained Mass	Calculated Mass	Deviation (ppm)			
Cyclophosphamide (CYP)	12.15 12.61	261.0330	261.0321	−3.4	C_7_H_15_Cl_2_N_2_O_2_P	[34,35]	
ID199	7.27	199.0386	199.0398	5.9	C_5_H_12_ClN_2_O_2_P	[34,35]	Elimination of chloroethyl group
ID275	10.53	275.0096	275.0114	6.4	C_7_H_13_Cl_2_N_2_O_3_P	[34,35]	Oxidation of the oxazaphosphinan group (possibly obtained from product ID277)
ID277	10.53	277.0212	277.0270	21.0	C_7_H_15_Cl_2_N_2_O_3_P	[34,36]	Hydroxylation of the oxazaphosphinan group
ID259	11.72	259.0150	259.0164	5.6	C_7_H_13_Cl_2_N_2_O_2_P	[35]	Elimination of H_2_O from product ID277

**Table 5:** Lamotrigine transformation products’ identification information.

N^o^	Rt [min]	[M+H]^+^ [Da]			Formula	Ref.	Suggested Occurrence
		Obtained mass	Calculated mass	Deviation (ppm)			
Lamotrigine (LMG)	10.12	256.0144	256.0151	2.9	C_9_H_7_Cl_2_N_5_	[38]	
ID270	3.12, 3.86	270.0369	270.0388	7.2	C_9_H_8_ClN_5_O_3_		Oxidation of amine group to nitro group
ID306	3.58, 3.77, 4.13, 4.32	306.0133	306.0155	7.3	C_9_H_9_Cl_2_N_5_O_3_		Two steps: Two hydroxylation of aromatic ring (ortho position) Obtains two carbonyl groups (one aldehyde and one carboxylic acid) after bond cleavage
ID238	5.09, 7.12	238.0487	238.0490	1.3	C_9_H_8_ClN_5_O	[38]	Exchange of chlorine atom with hydroxyl group—two possible substitution options
ID272	8.92, 9.85	272.0112	272.0100	−4.3	C_9_H_7_Cl_2_N_5_O	[38]	Hydroxylation of aromatic ring

**Table 6:** Valsartan transformation products’ identification information.

N^o^	Rt [min]	[M+H]^+^ [Da]			Formula	Ref.	Suggested Occurrence
		Obtained Mass	Calculated Mass	Deviation (ppm)			
Valsartan (Val)	15.93	436.2324	436.2343	4.4	C_24_H_29_N_5_O_3_	[40]	
ID366	13.81	366.15647	366.1561	−1.1	C_19_H_19_N_5_O_3_	[40]	Oxidation of methylene group and elimination of the pentanoyl group
ID452	14.54	452.2324	452.2292	−7.0	C_24_H_29_N_5_O_4_	[40]	Hydroxylation of aromatic ring (more than one option of each aromatic ring)
ID450	14.91	450.2141	450.2139	−1.2	C_24_H_27_N_5_O_4_	[40]	Oxidation of methyl group to aldehyde group

**Table 7:** Sulfamethoxazole transformation products’ identification information.

N^o^	Rt [min]	[M+H]^+^ [Da]			Formula	Ref.	Suggested Occurrence
		Obtained Mass	Calculated Mass	Deviation (ppm)			
Sulfamethoxazole (SMX)	11.41	254.0586	254.0594	3.1	C_10_H_11_N_3_O_3_S	[4,44]	
ID284	8.83, 9.98	284.0327	284.0336	3.1	C_10_H_9_N_3_O_5_S	[44,45]	Oxidation of amine group to nitro group
ID270	8.78, 10.86	270.0549	270.0543	−2.2	C_10_H_11_N_3_O_4_S	[44,45]	Hydroxylation of aromatic ring
ID99	4.26	99.0545	99.0553	8.0	C_4_H_7_N_2_O	[44,45]	Cleavage of N-S bond of the sulfonamide group

**Table 8:** Properties of the pharmaceutical compounds used in this study.

Name	Class	pKa *	Rate Constants		Ref.
			*k*O_3_ [M^−1^ s^−1^]	*k*_∙_OH [10^9^ M^−1^ s^−1^]	
Iohexol (IHX)	Contrast media	11.3	1.4	3.3	[21,48]
Bezafibrate (BZF)	Lipid regulator	3.3 (acid)	590	7.4	[21]
Lamotrigine (LMG)	Anticonvulsant	5.4 (base)	4	2.1	[38]
Valsartan (VAL)	Blood pressure	3.6, 4.2 (acid)	38	10	[49]
Cyclophosphamide (CYP)	Anticancer	2.84	2.8	1.3	[50]
Sulfamethoxazole (SMX)	Antibiotic	5.8 (acid)	4.7 × 10^4^ 5.7 × 10^5^	5.5, 8.5	[21,41,42]

* Obtained from ACDLABS, ACD/Percepta 2016.

**Table 9:** LC-MS chromatographic characteristics of the selected pharmaceutical compounds.

Compound	Iohexol (IHX)	Bezafibrate (BZF)	Lamotrigine (LMG)	Valsartan (VAL)	Cyclophosphamide (CYP)	Sulfamethoxazole (SMX)
RT (min)	4.4, 5.2	15.02	9.7	15.6	12.01	10.87
[MH]^+^	821.893	362.115	256.014	436.232	261.033	254.059

## Data Availability

Not applicable.

## Supplementary Materials

The following supporting information can be downloaded at: https://www.mdpi.com/article/10.3390/molecules28031227/s1, Table S1: Ecosar toxicity results for IHX; Table S2: Ecosar toxicity results for BZF; Table S3: Ecosar toxicity results for CYP; Table S4: Ecosar toxicity results for LMG; Table S5: Ecosar toxicity results for VAL; Table S6: Ecosar toxicity results for SMX.

## Sample Availability

Samples of the compounds are not available from the authors.
