# Supplementary material for: Pharmaceutical Transformation Products Formed by Ozonation—Does Degradation Occur?
Source: Molecules. 2023 Jan 26;28(3):1227. doi: 10.3390/molecules28031227 (PMC9919501; doi:10.3390/molecules28031227)
Supplement: Supplementary file 1 [file molecules-28-01227-s001.zip › molecules-2145859-supplementary.docx]

Supplementary Materials

**Table S1.** Ecosar toxicity results for CYP.

|  | **Fish, LC50 (mg/l)** | **Daphnia, LC50 (mg/l)** | **Fish, ChV (mg/l)** |
| --- | --- | --- | --- |
| CYP | 13 | 0.026 | 0.017 |
| ID 199 | 28 | 0.061 | 0.019 |
| ID 259 | 15.3 | 0.031 | 0.018 |
| ID 275 | 31 | 0.066 | 0.024 |
| ID 277 | 47.6 | 0.105 | 0.028 |

**Table S2.** Ecosar toxicity results for IHX.

|  | **Fish, LC50 (mg/l)** | **Daphnia, LC50 (mg/l)** | **Fish, ChV (mg/l)** |
| --- | --- | --- | --- |
| IHX | 1.29E+06 | 2.60E+06 | 2.05E+03 |
| ID 621 | 4.25E+05 | 7.97E+05 | 825 |
| ID 673 | 3.01E+04 | 4.49E+04 | 114 |
| ID 747 | 5.46E+05 | 1.03E+06 | 1.04E+03 |
| ID 819 | 1.01E+04 | 1.47E+04 | 1.60E+04 |
| ID 851 | 5.46E+06 | 1.02E+07 | 1.08E+04 |

**Table S3.** Ecosar toxicity results for VAL.

|  | **Fish, LC50 (mg/l)** | **Daphnia, LC50 (mg/l)** | **Fish, ChV (mg/l)** |
| --- | --- | --- | --- |
| VAL | 62.8 | 47.6 | 1.69 |
| ID 336 | 42.6 | 38.8 | 0.675 |
| ID 366 | 1.63E+03 | 1.65E+03 | 19 |
| ID 450 | 230 | 206 | 30 |
| ID 452 | 25.2 | 7.01 | 0.379 |

**Table S4.** Ecosar toxicity results for BZF.

|  | **Fish, LC50 (mg/l)** | **Daphnia, LC50 (mg/l)** | **Fish, ChV (mg/l)** |
| --- | --- | --- | --- |
| BZF | 17.6 | 12.2 | 0.618 |
| ID 290 | 2.18 | 0.515 | 0.031 |
| ID 292 | 5.11 | 0.759 | 0.06 |
| ID 318 | 0.379 | 0.233 | 0.019 |
| ID 368 | 242 | 476 | 16.3 |
| ID 394 | 11.5 | 4.57 | 0.198 |

**Table S5.** Ecosar toxicity results for LMG.

|  | **Fish, LC50 (mg/l)** | **Daphnia, LC50 (mg/l)** | **Fish, ChV (mg/l)** |
| --- | --- | --- | --- |
| LMG | 126 | 4.83 | 1.92 |
| ID 238 | 3.65E+01 | 1.65 | 0.269 |
| ID 270 | 4.97 | 0.718 | 0.058 |
| ID 272 | 2.19E+01 | 1.41 | 0.185 |
| ID 306 | 9.28E+00 | 8.25E+05 | 1.84 |

**Table S6.** Ecosar toxicity results for SMX.

|  | **Fish, LC50 (mg/l)** | **Daphnia,LC50 (mg/l)** | **Fish, ChV (mg/l)** |
| --- | --- | --- | --- |
| SMX | 267 | 6.43 | 5 |
| ID 99 | 270 | 3.63 | 6.59 |
| ID 270 | 35.9 | 1.75 | 0.273 |
| ID 284 | 322 | 353 | 2.99 |
